# Proteasome Subtypes and Regulators in the Processing of Antigenic Peptides Presented by Class I Molecules of the Major Histocompatibility Complex

**DOI:** 10.3390/biom4040994

**Published:** 2014-11-18

**Authors:** Nathalie Vigneron, Benoît J. Van den Eynde

**Affiliations:** 1 Ludwig Institute for Cancer Research, Brussels 1200, Belgium; E-Mail: benoit.vandeneynde@bru.licr.org; 2 WELBIO (Walloon Excellence in Life Sciences and Biotechnology), Brussels 1200, Belgium; 3 De Duve Institute, Université catholique de Louvain, Brussels 1200, Belgium

**Keywords:** antigenic peptides, proteasome, regulator, peptide splicing, processing, CTL

## Abstract

The proteasome is responsible for the breakdown of cellular proteins. Proteins targeted for degradation are allowed inside the proteasome particle, where they are cleaved into small peptides and released in the cytosol to be degraded into amino acids. In vertebrates, some of these peptides escape degradation in the cytosol, are loaded onto class I molecules of the major histocompatibility complex (MHC) and displayed at the cell surface for scrutiny by the immune system. The proteasome therefore plays a key role for the immune system: it provides a continued sampling of intracellular proteins, so that CD8-positive T-lymphocytes can kill cells expressing viral or tumoral proteins. Consequently, the repertoire of peptides displayed by MHC class I molecules at the cell surface depends on proteasome activity, which may vary according to the presence of proteasome subtypes and regulators. Besides standard proteasomes, cells may contain immunoproteasomes, intermediate proteasomes and thymoproteasomes. Cells may also contain regulators of proteasome activity, such as the 19S, PA28 and PA200 regulators. Here, we review the effects of these proteasome subtypes and regulators on the production of antigenic peptides. We also discuss an unexpected function of the proteasome discovered through the study of antigenic peptides: its ability to splice peptides.

## 1. Introduction to the Ubiquitin Proteasome System

The ubiquitin-proteasome system (UPS) is the major ATP-dependent protein degradation system in cells. It is essential to maintain cellular protein homeostasis and ensure the elimination of misfolded proteins (reviewed in [1,2]). Proteasome-dependent proteolysis is also essential to regulate a number of other cellular processes, such as cell differentiation, cell-cycle progression or apoptosis. Proteins targeted for degradation by the proteasome are tagged with polyubiquitin chains that are attached to lysine residues within the protein sequence. These ubiquitin-tagged proteins are recognized by the regulatory particle 19S (PA700), which is associated with the 20S proteasome catalytic core to form the 26S proteasome. Upon degradation, these ubiquitin-tagged proteins are degraded into small peptides of about three to 22 amino acids [3], which can be further degraded by cytosolic peptidases to recycle the pool of amino acids [4]. The immune system takes advantage of this system to monitor cellular integrity. Thus, a fraction of the peptides released by proteasomal degradation is transferred into the lumen of the endoplasmic reticulum (ER) by a dedicated transporter, named TAP (transporter associated with antigen processing) [5]. Inside the ER, those peptides will be further trimmed by ER-resident proteases, and peptides of an appropriate size (8–10 amino acids) and sequence will then associate with class I molecules of the major histocompatibility complex (MHC). Peptide-MHC complexes are finally displayed at the cell surface for potential recognition by cytolytic T-lymphocytes (CTLs). CTLs are major sentinels poised to rapidly recognize and destroy cells expressing mutant, infectious or tumoral proteins. It is the display of peptides (named antigenic peptides) derived from such altered proteins that marks cells for CTL recognition. The sensitivity of CTL, which can recognize target cells expressing as little as 10 molecules of antigenic peptide [6], enables the detection of only subtle changes in the cellular protein content. The detailed characterization of the antigenic peptides recognized by anti-tumor CTL that were isolated from the blood or tumor of cancer patients has provided valuable insights into the processing of antigenic peptides [7,8]. Such peptides usually derive from mutated proteins [9,10], from differentiation proteins [11,12] and from tumor proteins encoded by cancer-germline genes, such as the MAGE genes [13].

## 2. Role of the Proteasome in Antigen Processing

The involvement of the proteasome in the production of MHC class I peptides was originally stressed by the observation that the presentation of the model antigen, ovalbumin, was dependent on ubiquitination and, therefore, on the 26S proteasome [14,15]. In line with this, MHC class I presentation was accelerated when the N-terminus of protein antigens was genetically modified with a bulky or a charged residue to increase their rate of ubiquitination by the ubiquitin ligase E3 α (N-end rule) [16,17,18]. Another piece of evidence for the involvement of the proteasome in antigen processing came from the observation that proteasome inhibitors blocked the presentation of a number of antigenic peptides while limiting the overall peptide supply to MHC class I [19,20,21,22,23,24,25,26,27]. Although inhibitors reduced the cell ability to express specific antigenic peptides from full-length proteins, two studies showed that production of the antigenic peptides from minigenes of the correct size was not affected, suggesting that the inhibitors indeed specifically act on the peptide release from the full-length protein [19,28]. However, the role of proteasome inhibitors is often hard to interpret, first because inhibitors generally induce ER stress, which can affect the rate of synthesis of some proteins [29,30], and second, because they inhibit some catalytic activities of the proteasome, but not others. Their effect may therefore vary according to the main catalytic activity involved in the production of the peptide studied. In addition, the production of a number of peptides depends on a balance between productive cleavages, occurring mostly at the C-terminus, and destructive cleavages occurring within the sequence of the antigenic peptide [31,32]. The net effect of proteasome inhibitors on the production of such peptides therefore depends on the relative inhibition of the catalytic activities responsible for the productive *versus* destructive cleavages [33]. Studies on the presentation of constructs involving antigenic peptides extended at the C-terminus or the N-terminus indicated that the proteasome was usually required to produce the C-terminal cleavage, but was dispensable for the N-terminal cleavage [28,34]. Subsequent studies showed that N-terminally extended peptide precursors produced by the proteasome can be further trimmed by other peptidases, either in the cytosol by tripeptidyl peptidase II (TPPII) [35] or, more prominently, in the ER by the endoplasmic reticulum aminopeptidase ERAP1 [36,37]. Another strong argument for the role of the proteasome in the production of class I peptides lies in the observation that many antigenic peptides are differentially processed by the various proteasomes subtypes, as we will discuss below (Table 1) [31,32,38].

Although the proteasome is considered as the main source of antigenic peptides, it is now clear that it is not the only protease able to generate antigenic peptides [27,39]. Several alternative cytosolic proteases were shown to process specific antigenic peptides, including tripeptidyl peptidase II (TPPII) and the metallopeptidases thimet oligopeptidase, nardilysin and insulin degrading enzyme (IDE) [40,41,42,43,44]. However, the function of these proteases in general MHC class I presentation appears quite limited [45,46,47]. Additionally, antigenic peptides can also be produced by proteases present in the ER or in the secretory compartment. For example, signal peptidase and/or signal peptide peptidase are both involved in the production of TAP-independent peptides derived from signal sequences [48,49]. Moreover, in cells with a defect in peptide loading complex quality control, trans-Golgi proteases, such as protease pro-protein convertase 7, can rescue a significant proportion of the unstable MHC class I peptides, potentially by making peptides available from full-length proteins [50]. Coupling a murine cytomegalovirus (CMV) epitope to the C-terminus of the secretory hepatitis B protein, HBe, Gil-Torregrosa *et al.* showed that the trans-Golgi resident protease, furin, which is involved in HBe maturation along the secretory route, helped with releasing the CMV peptide for loading onto H-2L^d^ in TAP-deficient cells [51]. Finally, another alternative pathway involved in the presentation of MHC class I-restricted peptides is autophagy, a process that enables non-proteasomal degradation of proteins at steady-state and in conditions of stress, such as starvation or oxidative damage. In the MHC class I pathway, autophagy appears to contribute to the processing of a growing number of antigenic peptides presented by MHC class I and derived from endogenously expressed proteins [52,53,54]. In the autophagy-based MHC class I processing, cellular proteins are brought to the autophagosome/lysosomal compartment for degradation, and the peptides released are then loaded onto local MHC class I. This process is TAP-independent and gives rise to peptides that can be similar to those produced through the classical proteasome processing pathway [52,53,54]. Although the relative importance of autophagy in antigen processing still needs to be evaluated further, this process may be important to circumvent viral or tumoral evasion strategies affecting TAP transport. In this review, we will recapitulate the role played by the proteasome and its regulators in the processing of antigenic peptides.

**Table 1:** Processing efficiency of tumor antigens by the different proteasome types.

Peptide source	MHC restriction	Peptide Sequence	Standard Proteasome	Immuno-proteasome	Intermediate Proteasome β5i	Intermediate Proteasome β1i–β5i	References
**Self**							
RU1_34–42_	HLA-B51	**VPYGSFKHV**	+ + ^§^	+/−	n.d.	n.d.	[20,31]
FGF-5_172–176__ and__ 217–220_ *	HLA-A3	**NTYAS_PRFK**	+ +	−	n.d.	n.d.	[55,56]
**Differentiation**							
gp100_40–42__ and__ 47–52_ *	HLA-A32	**RTK_QLYPEW**	+ +	+/−	n.d.	n.d.	[22,56]
gp100_209–217_	HLA-A2	**ITDQVPFSV**	+ +	+/−	+/−	+/−	[12,31,38]
gp100_195–202__ and__ 191 or 192_ *	HLA-A3	**RSYVPLAH_R**	+ +	n.d.	n.d.	n.d.	[57]
Tyrosinase_369−377_	HLA-A2	**YMDGTMSQV**	+ +	+/−	−	+/−	[31,38,58]
Tyrosinase_368–373 and 336–340_ *	HLA-A24	**IYMDGT_ADFSF**	+ +	−	n.d.	n.d.	[59]
Melan-A_26–35_	HLA-A2	**EAAGIGILTV**	+ +	−	+/−	+/−	[38,60]
**Cancer germline**							
MAGE-A3_114–122_	HLA-B40	**AELVHFLLL**	−	+ +	+ +	+ +	[31,38,61]
MAGE-A3_271–279_	HLA-A2	**FLWGPRALV**	−	−	+ +	−	[32,62]
MAGE-A10_254–262_	HLA-A2	**GLYDGMEHL**	−	−	−	+ +	[32,63]
MAGE-C2_336–344_	HLA-A2	**ALKDVEERV**	−	+ +	−	+ +	[31,38,64]
MAGE-C2_42–50_	HLA-B57	**ASSTLYLVF**	−	+ +	+ +	+ +	[38,65]
MAGE-C2_191–200_	HLA-A2	**LLFGLALIEV**	−	−	−	+ +	[38,64]
**Mutation**							
CLPP_240–248_	HLA-A2	**ILDKVLVHL**	+/−	+ +	n.d.	n.d.	[66]
HAUS3_154–162_	HLA-A2	**ILNAMIAKI**	−	n.d.	n.d.	+ +	[67]
**Polymorphism (mHC ^||^)**							
SP110_296–301 and 286−289_ *	HLA-A3	**SLPRGT_STPK**	+/−	+ +	n.d.	n.d.	[56,68]

* Spliced peptide. ^§^ + +, efficiently produced; +/−, slightly produced; −, not produced; n.d., not determined. ^||^ mHC, minor histocompatibility antigen.

## 3. The 20S Proteasome

**Figure 1:**
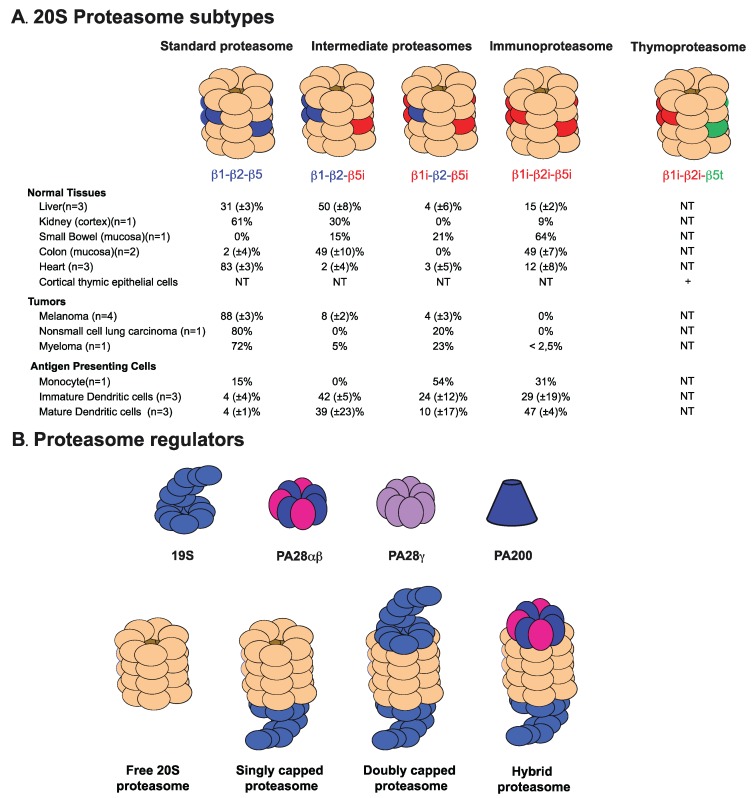
Proteasome subtypes and regulators. (**A**) Mammalian proteasomes are composed of a 20S core particle, made of four stacked rings of seven subunits each. The two outer rings are made of α subunits, and the two inner rings are made of β subunits, three of which (β1, β2, β5) are catalytically active. Upon induction with IFNγ or in immune cells, these catalytic subunits are replaced with their inducible counterparts, β1i, β2i, β5i, to form immunoproteasomes. Besides standard and immunoproteasomes, two additional forms of proteasome exist, which contain a mixture of standard and immune catalytic subunits, as indicated. The thymic cortex expresses a specific catalytic subunit, called β5t, which combines with β1i and β2i to form the thymoproteasome. The percentage of each proteasome type as measured by a sandwich ELISA approach [32] is indicated for different tissue and tumor types. Thymoproteasome is specifically expressed in cortical epithelial cells (cortical thymic epithelial cells (cTEC)). (**B**) The two α-rings of the 20S proteasome interact with regulatory particles of four different types: PA700 (19S), PA28αβ, PA28γ and PA200. These regulatory particles can bind to one or both sides of the 20S particle or form hybrid proteasomes where the 20S core binds two different regulators. Representative examples are shown. NT, not tested.

**Figure 2:**
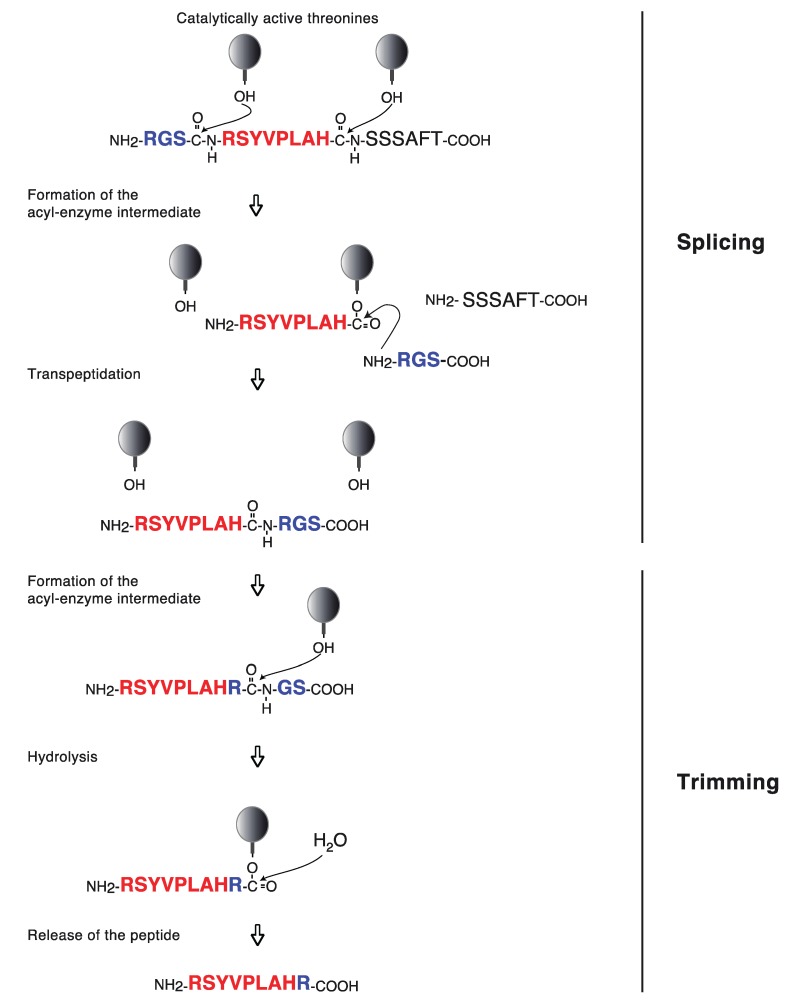
Peptide splicing and trimming of the antigenic peptide RSYVPLAH_R derived from gp100 by the proteasome. In the course of peptide-bond hydrolysis, the hydroxyl group of the N-terminal threonine produces a nucleophile attack on the carbonyl of the peptide bond. This leads to the formation of an acyl-enzyme intermediate in which the peptide fragment RSYVPLAH remains attached to the proteasome through an ester link. Normally, this acyl-enzyme intermediate is rapidly hydrolyzed. However, peptide fragments, such as RGS, present in the chamber can compete with water molecules, their free N-terminal amino-group performing a nucleophilic attack on the acyl-enzyme intermediate. This transpeptidation leads to the creation of a new peptide bond, which assembles both fragments, leading to the formation of a spliced peptide bearing an extended C-terminus. The C-terminal extension is then further trimmed by the proteasome to release the antigenic peptide, RSYVPLAHR. Balls represent the catalytic β subunits of the proteasome. The hydroxyl group of the N-terminal threonine is indicated.

The 20S proteasome is a cylindric particle of about 700 kDa, which is composed of four stacked heptameric rings, each comprising seven subunits. The two outer rings are identical and made of structural α subunits, and the two inner rings each contain seven β subunits, three of which mediate the catalytic activity of the proteasome: β1, β2 and β5 (Figure 1A). Mutagenesis studies and the analysis of the crystal structure of proteasomes complexed with inhibitors showed that peptide bond hydrolysis is mediated by the N-terminal threonine of the catalytically-active β subunits, which is exposed to the luminal side of the proteasome particle, in the catalytic chamber [69,70,71,72]. The hydroxyl group of the side chain of this threonine produces a nucleophilic attack on the carbonyl of the peptide bond, leading to the formation of an acyl-enzyme intermediate in which a peptide fragment remains attached to the proteasome by an ester link. Water molecules present in the chamber rapidly hydrolyze this acyl-enzyme intermediate, releasing a peptide fragment that is then transferred back into the cytosol [73]. A few years ago, we found that the proteasome was also able to produce antigenic peptides by creating a peptide bond between peptide fragments originally distant in the parental protein [22]. So far, five antigenic peptides were shown to be produced by this new function of the proteasome, called peptide splicing (Table 1) [22,55,57,59,68,74]. We showed that peptide splicing takes place in the proteasome chamber, through a process of transpeptidation that involves the nucleophilic attack of the acyl-enzyme intermediate by the N-terminus of a peptide fragment present in the catalytic chamber (Figure 2) [22]. Interestingly, among the five spliced peptides identified to date, three were composed of two non-contiguous peptide fragments spliced together in the reverse order to that in which they occur in the parental protein [57,59,68]. Given their nature, spliced antigenic peptides therefore appear as byproducts of the normal proteasome degradation activity. This process leads to the production of a large variety of peptides, as peptide splicing could theoretically involve any peptide fragment released by proteolysis, provided that the nucleophile peptide contains at least three amino acids [57]. Studying the peptide RSYVPLAH_R (splicing of fragment RSYVPLAH with a single arginine) derived from gp100 and presented by HLA-A3, we showed that this minimal size requirement for the nucleophile peptide resulted in the production of a spliced peptide bearing an extended C-terminus, which is then further trimmed by the proteasome in order to produce the final antigenic peptide (Figure 2) [57]. Recently, Mishto *et al.* suggested the existence of an additional pocket that would accommodate the nucleophile peptide and would be distinct from the primed substrate binding site, which accommodates the part of the peptide substrate located C-terminally from the cleavage site [75]. They suggested that this additional pocket might already be occupied by the splice reactant prior to the formation of the acyl-enzyme intermediate, thereby facilitating and speeding up the splicing reaction. This suggestion arose from their observation that the hierarchy of splicing at various sites within a given peptide did not strictly follow the hierarchy of cleavage at those sites, suggesting additional constraints determining splicing efficiency, such as the affinity of the nucleophile peptide for this postulated pocket.

So far, the identification of spliced antigenic peptides has been limited by the availability of specific CTL directed against these peptides. Using a specific computer-based algorithm applied to the mass spectrometry analysis of peptide digests, Liepe *at al.* developed a novel approach (SpliceMet) to predict spliced antigenic peptides [76]. One could verify the relevance of these peptides by isolating CTL *in vitro* using the reverse immunology approach [77]. The presence of the predicted peptide at the surface of target cells would then be confirmed by analyzing the ability of these cells to activate the corresponding CTL.

In essence, the proteolytic activity of the proteasome is not specific: any protein or peptide entering the proteasome can be degraded. It is therefore essential for cellular homeostasis to tightly control the activity of such an abundant intracellular protease, so as to prevent undesired degradation of intracellular proteins. The proteasome therefore evolved as a compartmentalized particle delineating a chamber that is not accessible to surrounding proteins and that contains the catalytically active sites [78]. This enables the control of proteasome activity at the level of the entry of substrates in the catalytic chamber. Indeed, regulators of proteasome activity, such as the 19S regulator, function as gate-keepers, allowing entry into the proteasome particle of only those substrates that need to be degraded and preventing entry of others. Entrance to the chamber is first limited by an aperture consisting of a 13-Å pore delimited by the ring formed by the α subunits, as observed in the *Thermoplasma* proteasome [69]. The small size of this α-annulus ensures that only unfolded proteins can enter the catalytic chamber. Additionally, the entrance of the chamber is further obstructed in yeast and bovine proteasome by the N-terminal tails of the α subunits that form a tight polypeptide net, which is projected inside the pore and prevents access of smaller substrates to the catalytic chamber [70,79]. Free 20S proteasome therefore has a low activity, at least toward folded protein substrates [2]. Opening of the α-gate is tightly regulated through the binding of specific activator complexes, called regulatory particles, which are believed to interact with the N-terminal tails of the α-subunits, inducing their displacement and the opening of the gate [2]. Four types of regulators exist: the 19S regulator (PA700), the 11S regulators, PA28αβ and PA28γ, and the PA200 regulator, whose functions will be discussed below.

As an exception to this rule of access-control by regulators, it has been shown that unfolded protein substrates and oxidant-damaged proteins can be degraded by free 20S proteasomes [80,81]. A large excess of enzymatically-active, free 20S proteasome over 26S proteasome is indeed found within cells [82,83]. A recent study from Baugh *et al.* suggested that about 20% of total cellular proteins can be degraded by the 20S proteasome [84]. However, how unstructured proteins or oxidant-damaged proteins enter the catalytic core of the 20S chamber is still not well understood. Various studies suggested that access to the catalytic chamber could be controlled by the substrate itself or facilitated by accessory proteins, such as Hsp90 [85] or arginine-rich histones [86]. It has been suggested that hydrophobic patches that are often exposed after protein oxidation might facilitate the entry of oxidized proteins into the catalytic chamber [80], in line with the fact that hydrophobic peptides appear to promote the opening of the α-ring of the 20S proteasome [87]. Conditions of stress, such as oxidative damage or prolonged starvation, induce 26S proteasomes to dissociate into 20S proteasomes and 19S sub-complexes [88,89,90]. This leads to a rapid decrease in proteolysis and an accumulation of ubiquitinated proteins, but could favor the degradation of poorly-structured or oxidized proteins by 20S proteasomes [88,91]. Reports have suggested that PA28αβ could also assist the 20S proteasome in the degradation of oxidized proteins [81,92].

## 4. Regulation of the Proteasome Activity

The first level of regulation is achieved by changes in the structure of the 20S proteasome, which exists in several isoforms or subtypes, having different catalytic activities.

### 4.1. Proteasome Subtypes

#### 4.1.1. Standard Proteasome and Immunoproteasome

In immune cells or after exposure to the inflammatory cytokines, IFN-γ or TNF-α, alternative catalytic subunits, named LMP2 (β1i), MECL-1 (β2i) and LMP7 (β5i), are expressed and incorporated into the proteasome in place of their constitutive counterparts, β1, β2 and β5, to form another particle called the immunoproteasome (Figure 1A) [93]. Based on the degradation of small fluorogenic peptides, three major catalytic activities of the proteasome were described, the caspase-like, trypsin-like and chymotrypsin-like activities, which cleave after acidic, basic and hydrophobic residues, respectively. The study of yeast proteasome mutants suggested that each of these catalytic activities was associated with one specific catalytic subunit. The caspase-like activity is linked to β1, while the trypsin-like and chymotrypsin-like activities are associated with β2 and β5, respectively [72,94,95]. However, it is now clear that the catalytic specificity is more complex than originally expected, since some proteasome subunits have overlapping specificities [95], and the position of the cleavage site can also be influenced by the surrounding residues [96,97]. Nevertheless, the immunoproteasome activity, as measured with fluorogenic peptides, is characterized by a lower caspase-like activity and higher trypsin-like and chymotrypsin-like activities [98,99]. Due to these differences in their catalytic sites, the standard proteasome and the immunoproteasome produce distinct peptide sets, which only partly overlap [97,98,100,101]. Because the immunoproteasome shows an increased propensity to cleave after basic or hydrophobic residues, it was predicted to be more efficient at producing antigenic peptides with high affinity for MHC class I molecules, which often call for peptides bearing basic or hydrophobic residues at their C-termini [98,100]. This idea was corroborated by the study of knockout mice for either β1i or β5i, in which the presentation of several epitopes was decreased [102,103]. This was recently confirmed using knockout mice for all three immunosubunits, whose dendritic cells fail to present a number of MHC class I epitopes and whose splenocytes display a peptide repertoire that is about 50% different from that of wild-type cells [104]. In the same line, the peptide repertoire of dendritic cells from β2i^−/−^β5i^−/−^ mice is less abundant and diverse than that of wild-type mice [105]. Overall, these results show that the immunoproteasome plays an important role in MHC class I antigen presentation. Several human tumor antigens are also better produced by the immunoproteasome than by the standard proteasome (Table 1) [31,32,38,61,65]. However, it is also clear that a number of antigenic peptides are better produced by the standard proteasome. The first example of such a peptide was described by Morel *et al.* who studied a human CTL clone recognizing a peptide derived from ubiquitous protein RU1. This peptide was presented by kidney cancer cells, but not by the autologous EBV-B cells [20]. This was explained by the fact that the peptide was processed efficiently by the standard proteasome, which was present in kidney cancer cells, but not by the immunoproteasome, which was abundant in the EBV-transformed B-cells. Other peptides that are efficiently processed by the standard proteasome, but not by the immunoproteasome, were subsequently identified (Table 1) [31,106]. *In vitro* digestions of peptide precursors with purified proteasomes indicated that the lack of processing by one proteasome type generally resulted from a prominent cleavage within the antigenic peptide, resulting in its destruction [31,32,38]. Similar observations were made when Basler *et al.* studied the immunoproteasome-dependent generation of the murine minor histocompatibility antigen UTY_246–254_ and the human influenza matrix peptide_58–66_ [107]. The authors showed that inhibition of the β1 or the β5 subunit, respectively, rescued the presentation of these peptides, suggesting that the standard subunits were responsible for their degradation.

In the past few years, we and others identified several peptides produced by peptide splicing in the proteasome. As described above, the peptide splicing reaction takes place in the proteasome trough a reaction of transpeptidation involving the nucleophilic attack of an acyl-enzyme intermediate by another peptide fragment present in the chamber [22]. We found that three spliced peptides were better produced by the standard proteasome than by the immunoproteasome, while another was more efficiently produced by the immunoproteasome [56]. This demonstrated that both proteasome types can perform the splicing reaction. However, we observed that the production of a given spliced peptide depended on the ability of the proteasome to perform the cleavages required to liberate the fragments that composed the spliced peptide. Additionally, this ability differed among the two proteasome subtypes. Of note, even though it was initially identified in tumor cells, peptide splicing is not restricted to tumor cells, but can also occur in non-tumor cells, such as melanocytes [59]. *In vitro*, it was produced using proteasomes originating from human erythrocytes, but also from yeast cells [22,75]. It is therefore expected that peptide splicing also occurs in the thymus, where spliced peptides should be expressed like any other self-peptides to establish natural immune tolerance, on the condition that the type of proteasome (standard or immunoproteasome) present in medullary thymic epithelial cells is adequate for the production of these peptides.

The induction of T-cell immune responses is critically dependent on dendritic cells, which are professional antigen-presenting cells located mostly in lymph nodes. These cells uniquely express the set of cytokines and costimulatory molecules required to activate naive T-cells in an antigen-specific manner. This means that CTL responses will be induced only against antigenic peptides presented by dendritic cells. Dendritic cells contain immunoproteasomes and intermediate proteasomes (see below), but very little standard proteasome (Figure 1A) [32]. Therefore, they will mostly induce CTL responses against peptides produced by the immunoproteasome (or intermediate proteasomes), but not against peptides produced by the standard proteasome. Regarding anti-viral responses, this is not an issue, because viral infections usually trigger inflammation, which induces immunoproteasomes in infected cells, which therefore will present the adequate antigenic peptides. Indeed, immunoproteasome-dependent peptides were shown to be the dominant targets of anti-viral CTL responses [108]. However, in the case of anti-tumor immune responses, the situation is different, because tumor cells usually do not contain immunoproteasomes, unless they are exposed to inflammation, and therefore, they may not present the peptides against which the immune response was triggered by dendritic cells [106]. Moreover, as indicated above, a number of clinically relevant tumor antigens are produced by the standard proteasome, but not by the immunoproteasome, and therefore are not presented efficiently by dendritic cells [20,31]. Vaccination strategies aimed at inducing CTL against such antigens should therefore be adapted in order to by-pass processing by the proteasome of dendritic cells. This can be done by immunizing with short “pre-processed” synthetic peptides or with recombinant minigene constructs encoding only the short peptide. This notion was first confirmed by a study comparing the CTL responses induced against the HLA-A2-restricted peptide, melan-A_26-35_, after vaccination of wild-type and β1i-knock-out HLA-A2 transgenic mice with either the peptide-encoding minigene or the full-length melan-A [109]. Stronger responses were induced in wild-type mice when the immunization was performed using the minigene retroviral construct when compared to the full-length construct, and this difference was reduced in the β1i-knockout mice, which developed stronger responses to the full-length construct than the wild-type mice. This confirmed that the immunoproteasome was responsible for the poor responses in wild-type mice. Another approach to induce CTL responses against standard proteasome-dependent peptides is to engineer dendritic cells so that they no longer contain immunoproteasomes, but only standard proteasomes. Dannull *et al.* successfully used this approach in humans, using siRNA for the catalytic immunosubunits. They showed that dendritic cells transfected with those siRNA and with RNA encoding full-length tumor antigens induced better anti-tumor CTL responses, not only *in vitro*, but also *in vivo* in melanoma patients [110,111].

As mentioned above, the immunodominance hierarchy of viral antigens is linked to their dependency on processing by the immunoproteasome. Indeed, studying CTL responses against various influenza, MCMV (mouse cytomegalovirus) and LCMV (lymphocytic choriomeningitis virus) peptides, several groups observed changes in the responses to these peptides in knockout mice for some of the immunoproteasome subunits [108,112,113,114,115]. Responses to antigenic peptides were generally decreased, but in some cases, responses were improved or remained stable. Interestingly, this ended up modifying the immunodominance hierarchy, with dominant epitopes becoming subdominant, and *vice versa* [108]. The modification of the responses observed in these different models were generally attributed to a difference in the processing of the antigenic peptide by the proteasome types and/or to an alteration of the T-cell repertoire in immunoproteasome-deficient animals [108,112,114]. This latter point implies that, in immunoproteasome-deficient animals, the negative selection operated in the thymus has led to the deletion of T-cell precursors recognizing those viral epitopes. Negative selection occurs in the thymic medulla, where medullary thymic epithelial cells (mTEC) present MHC class I-restricted peptides derived from a variety of self-proteins. This induces the selective deletion of T-cell precursors reactive to these peptides. The proteasome content of mTEC has not yet been clearly evaluated, but it is thought to mostly correspond to immunoproteasomes. Alteration of the anti-viral repertoire in immunoproteasome subunit-deficient animals therefore suggests that the absence of some immunoproteasome subunits in mTECs enables the expression of novel self-peptides that mimic the viral epitopes and lead to the deletion of the corresponding anti-viral T-cell precursors.

Studying the role of the immunoproteasome in the establishment of a T-cell response to MCMV in wild-type and β5i^−/−^ mice, Hutchinson *et al.* observed that although acute responses to MCMV mostly targeted immunoproteasome-dependent peptides, memory responses targeted those two epitopes that showed the least dependency on the immunoproteasome [115]. Acute MCMV T-cell responses would therefore be elicited by cells containing some immunoproteasome. However, this response would not be totally efficient at destroying target cells infected by MCMV, as this virus impedes immunoproteasome formation through the action of M27, a protein that induces the proteasomal degradation of STAT2 and thereby inhibits the IFNγ response pathway [116]. Chronic responses to MCMV would then involve antigen-presenting cells containing only standard proteasomes and stimulating CTL against those epitopes that are the least dependent on the immunoproteasome.

#### 4.1.2. Intermediate Proteasomes

Using a unique set of antibodies directed against proteasome catalytic subunits, our group additionally described two alternative proteasome forms, which are intermediate between the standard and the immunoproteasome, and contain only one (β5i) or two (β1i, β5i) of the three catalytic subunits of the immunoproteasome (immunosubunits) (Figure 1A) [32]. The existence of intermediate proteasome forms had previously been inferred from the observation that a number of tissues contained only some of the three immunosubunits [117]. It is only the development of immunosubunit-specific antibodies able to capture proteasomes in their native form that allowed us to define the exact stoichiometry of the intermediate proteasomes that are found in human tissues [32]. The existence of only two forms of intermediate proteasomes, namely β5i and β1i–β5i, is in agreement with the previous works dissecting the general rules for proteasome cooperative assembly [93,118], which is dependent on the nature of the subunit propeptides [119,120]. This cooperative assembly ensures a preferential incorporation of immunosubunits over standard subunits, where, e.g., incorporation of β2i is dependent on that of β1i [93]. This may explain why we did not detect the presence of intermediate proteasomes containing β2i. In addition, catalytic subunits are initially incorporated in an immature form, containing an N-terminal peptide that needs to be cleaved to liberate the N-terminal threonine of the mature active site [121]. This maturation step occurs at the last stage of proteasome assembly, when two hemiproteasomes join together to form the final particle. Again, this ensures protection of the cellular proteins, as catalytic sites only become active when the final particle is assembled and confines the proteolytic activity to the inner chamber. Maturation of the immunosubunits is interdependent: β5i was shown to be required for the maturation of β1i and β2i [93]. This may explain why we did not detect intermediate proteasomes lacking β5i. Because the proteasome particle contains two β rings, the existence of proteasomes containing an asymmetric assortment of immunosubunits and standard subunits is conceivable. Such asymmetric proteasomes were observed in cells transfected with a tagged β1 and exposed to IFNγ [122], but it remains unclear whether they exist in unmanipulated cells or tissues. The intermediate proteasomes we described were symmetric. We searched for asymmetric proteasomes containing β5 on one β ring and β5i on the other, and we did not find any in human liver and melanoma [32].

Interestingly, intermediate proteasomes β5i and β1i–β5i represent 10% to 20% of the total proteasomes found in tumors and 30% to 50% of those found in liver, kidney, small bowel, colon and dendritic cells [32] (Figure 1A). Studying the specific cleavage properties of each proteasome type, we showed that intermediate proteasomes β5i and β1i–β5i both display trypsin and chymotrypsin-like activities that are intermediate between those of the standard and the immunoproteasome. The caspase-like activity of intermediate proteasome β5i is similar to that of the standard proteasome, while this activity is low in intermediate proteasome β1i–β5i. This is easily explained by the fact that the caspase-like activity is generally associated with the β1 subunit, which is present in the β5i intermediate proteasome and not in the β1i–β5i intermediate proteasome [32]. Owing to their specific cleavage properties, intermediate proteasomes produce a unique set of antigenic peptides. The HLA-A2-restricted peptides, MAGE-A10_254–262_ and MAGE-C2_191–200_, are exclusively produced by the intermediate proteasome, β1i–β5i, while the HLA-A2-restricted peptide, MAGE-A3_271–279_, is processed only by intermediate proteasome β5i (Table 1) [32,38]. Hence, intermediate proteasomes enlarge the MHC class I repertoire to peptides that can be processed by both dendritic cells (containing immunoproteasomes and intermediate proteasomes) and tumor cells (containing standard proteasomes and intermediate proteasomes) and might therefore represent valuable targets for cancer immunotherapy. Of note, the presence of intermediate proteasomes should also be taken into account when analyzing the role of the immunoproteasome in single-immunosubunit KO mice.

#### 4.1.3. Thymoproteasome

A few years ago, Murata *et al.* discovered the existence of an additional proteasome catalytic subunit, called β5t, which is specifically expressed in the cortical thymic epithelial cells (cTEC) and is homologous to the β5 and β5i subunits [123,124]. The cTEC are responsible for positive selection, a process by which immature T-cell precursors able to bind to self-MHC class I/peptide complexes on cTEC are triggered to survive. The β5t subunit has a dramatic effect on the establishment of the CTL repertoire, as β5t-deficient mice display an 80% reduction of splenic CTL and do not survive influenza virus infection [123,125]. The β5t subunit is incorporated in the 20S proteasome instead of β5i along with β1i and β2i (Figure 1A) [123]. Incorporation of β5t into HEK-293T proteasomes induced a dramatic decrease of the chymotrypsin-like activity when compared to the β5-containing proteasome, while the trypsin-like and caspase-like activities were not affected [123]. This is in line with the fact that the S1 pocket of β5t is lined by hydrophilic amino acids, while that of β5 or β5i contains hydrophobic residues [123]. Using a unique set of antibodies recognizing peptide-bound or empty MHC class I molecules, Nitta *et al.* showed that cTECs from both wild-type or β5t^−/−^ mice express MHC class I molecules associated with peptides. Some of these peptides appeared unique to cTECs, as two specific peptide populations bound to H-2K^b^ were found to be differentially expressed in cTECs when compared to other APCs [125], suggesting that cTECs display a qualitatively distinct repertoire of MHC class I/peptide complexes. It was originally proposed that the low chymotrypsin-like activity of the thymoproteasome might produce low-affinity MHC class I ligands that may limit the duration or the avidity of the interaction of the peptide/MHC complexes with the TCR and facilitate positive selection [123,126]. However, recent work by Xing *et al.* showed that the half-life of MHC/peptide complexes on the surface cTEC and mTEC was similar, disagreeing with the idea that cTEC would carry unstable MHC complexes responsible for positive selection [127]. Moreover, similar surface MHC class I levels are found in β5t^+/−^ and β5t^−/−^ cTECs [123]. To reconcile these observations, it was proposed that cTECs carried peptides binding MHC class I using alternative anchor positions or developed a mechanism enabling them to stabilize MHC class I molecules bearing low affinity peptides [128]. In the future, a more detailed analysis of the peptide repertoire found on cTECs might help to understand the role played by the thymoproteasome in the formation of an immunocompetent T-cell repertoire.

#### 4.1.4. Proteasome Subtypes and Negative Selection of T-Cell Precursors in the Thymus

It is interesting to note that, so far, most tumor antigenic peptides found to be poorly processed by the immunoproteasome derive from self-proteins expressed in normal adult tissues (Table 1). Because the thymus contains abundant amounts of immunoproteasome, this precludes the processing of these peptides by the medullary thymic epithelial cells (mTEC), resulting in a lack of negative selection of T-cells recognizing these antigens and explaining why these T-cells persist in the periphery. In contrast, tumor antigens, which are better produced by the immunoproteasome, often correspond to MAGE-type antigens encoded by cancer-germline genes or to mutated antigens [31,32,66]. The limited immune tolerance against MAGE-type antigens has been attributed to the very low expression of cancer-germline genes in mTEC, resulting in the negative selection of only those T-cells recognizing these antigens with a high avidity [129]. The anti-tumor T-lymphocytes present in the blood of cancer patients would then derive from low-avidity T-cells that escape negative selection in the thymus. To confirm this concept, our group recently produced knockout mice for a mouse cancer-germline gene, namely P1A [130]. The expectation was that these mice, which are devoid of P1A expression in the thymus, should contain an unselected anti-P1A T-cell repertoire still containing high-affinity T-cells. Surprisingly, although the frequency of anti-P1A T-cells was slightly higher in P1A-KO mice, the anti-P1A T-cell repertoire of P1A-KO mice was barely different from that of wild-type mice, indicating that there is only minimal tolerance against this antigen in wild-type mice, despite detectable expression of P1A in mTEC [130]. Further analysis of the processing of the P1A antigen and of the type of proteasome contained in mTEC might help understanding the reason for the lack of tolerance against antigens encoded by cancer-germline genes.

#### 4.1.5. Structure-Related Properties

The crystal structures of the mouse standard proteasome and the immunoproteasome were solved recently and revealed that the cleavage preferences of the immunoproteasome relied on subtle conformational changes in the substrate-binding pockets of catalytic subunits β1i and β5i [131]. Indeed, the substrate-binding channel of β1i is lined with hydrophobic amino acids, resulting in an increased hydrophobicity and a decreased size of the binding pocket, thereby accounting for the higher cleavage activity of β1i after small hydrophobic and branched residues [131]. Compared to β5, β5i harbors a more spacious S1 pocket, favoring access to large non-polar residues and explaining the higher chymotrypsin-like activity of β5i [131]. Additionally, the vicinity of the active site of β5i is more hydrophilic: this is predicted to favor proteolysis by attracting water molecules, but also by stabilizing the tetrahedral intermediate through additional hydrogen bonds with the oxyanion hole [131].

Studying the processing of the HLA-A2-restricted peptide, MAGE-A10_254–262_, we observed that this peptide could only be processed by the intermediate proteasome β1i–β5i and that the inability of the other proteasomes to produce the peptide was related to the occurrence of a destructive cleavage within the peptide after aspartate_257_ (D_257_) (Table 1) [32]. The caspase-like activity, which is defined by cleavage after acidic residues, is generally associated with the presence of the β1 subunit, and digestion of fluorogenic peptides showed that the intermediate proteasome β1i–β5i and the immunoproteasome display a much lower caspase-like activity than the standard proteasome. However, in the case of the MAGE-A10_254–262_ peptide, the immunoproteasome still performed the destructive cleavage after D_257_, despite the presence of the β1i subunit. Therefore, the ability of the intermediate proteasome β1i–β5i, as opposed to the immunoproteasome, to produce the antigenic peptide suggests that β2i negatively influences the production of this peptide, either because β2i itself displays sufficient caspase-like activity to destroy the peptide or because it affects the structure of the immunoproteasome and increases its ability to cleave after D_257_. Such an allosteric effect of one subunit on the activity of the others was previously suggested by Sijts *et al.* who showed that subunit β5i, by impacting the structure of the 20S proteasome, increases the activity of subunits β2i and β1i [132].

#### 4.1.6. Other Functions of the Immunoproteasome

It is quite likely that the function of the different proteasome types is not restricted to antigen processing. The work of Seifert *et al.*, for example, suggested a role for the immunoproteasome in the degradation of polyubiquitinated, oxidant-damaged proteins accumulating after IFNγ treatment [133]. However, the prominent role of the immunoproteasome in the degradation of polyubiquitinated proteins was not confirmed by others [134]. Another role for the immunoproteasome was suggested by the observation that the absence of β1i in NOD mice impaired the processing of the NF-κB p105–p50 [135]. However, this study was refuted early after publication, and the function of the immunoproteasome in the NF-κB signaling pathway has remained largely debated since then [136,137,138,139,140]. Interestingly, the use of the β5i-specific inhibitor, PR-957, affected the production of inflammatory cytokines IL-23, TNF-α and IL-6 by endotoxin-stimulated peripheral blood mononuclear cells in an NF-κB-independent manner [141]. The inhibitor also affected the production of IFNγ and IL-2 in stimulated T-cells and helped with controlling the pathogenic response in a mouse model of rheumatoid arthritis [141].

### 4.2. Proteasome Regulators

As mentioned above, a key step in the control of proteasome activity is exerted at the level of substrate entry into the catalytic chamber of the particle and depends on the presence of regulators.

#### 4.2.1. The 19S Regulator

The 19S regulator (PA700) is a large particle of about 700 kDa that associates with one or both sides of the 20S proteasome core to enable the proteasome to bind, deubiquitylate, unfold and translocate the protein substrate into the catalytic chamber. The 19S regulator specifically recognizes proteins targeted for degradation by the covalent attachment of ubiquitin molecules (74 amino acids in length) to lysine residues within protein substrates. The ubiquitination requires the sequential action of an ubiquitin-activating enzyme, E1, an ubiquitin-conjugating enzyme, E2, and an ubiquitin-ligase enzyme, E3 (reviewed in [142]). Additional ubiquitin molecules can then be ligated to a previously bound ubiquitin to form polyubiquitin chains. Chains of four or more ubiquitins are sufficient to target proteins for degradation.

The 19S regulator is composed of at least 19 subunits that are assembled in two different parts: the lid and the base (reviewed in [2]). In yeast, the base comprises six ATPases (Rpt1-6), the two large non-ATPase subunits, Rpn1 and Rpn2, and two ubiquitin receptor subunits, Rpn10 and Rpn13. The six AAA-ATPase subunits, Rpt1-6, form a heterohexameric ring that directly contacts the α ring of the core 20S particle and is responsible for the ATP-dependent unfolding and translocation of the substrate into the chamber. The Rpn10 and Rpn13 subunits directly bind the polyubiquitin chains of protein substrates. The lid comprises nine Rpn subunits (Rpn3, 5–9, 11, 12 and 15). Subunits Rpn3, 5, 6, 7, 9, 12 have very similar domain structures and comprise an N-terminal helix repeat segment, a proteasome-COP9/signalosome-eIF3 (PCI) domain and a long C-terminal helix [143]. The Rpn8 and 11 subunits contain an Mpr1-Pad1-N-terminal (MPN) domain, and Rpn11 bears a deubiquitylation activity (DUB) [144,145]. The role of the other PCI and MPN subunits has not yet been characterized, except for Rpn6, whose crystal structure suggested a critical role in stabilizing the interaction between the 20S core particle and the regulatory complex [146]. The 19S regulator can associate with one or both ends of the 20S proteasome particle, but can also be part of a hybrid proteasome composed of one 19S regulator and one PA28 or PA200 regulator bound to either end of the 20S proteasome complex (Figure 1B) [147,148].

#### 4.2.2. The PA28αβ Regulator

The PA28αβ regulator (11S regulator) was originally isolated owing to its ability to stimulate the hydrolysis by the 20S proteasome of small peptide substrates, but not intact, folded proteins [149,150,151]. It is composed of two subunits, α and β, whose transcription is induced following exposure to IFNγ [152,153]. Electron-microscopic studies showed that the PA28αβ subunits assemble to form a ring-shaped heptameric complex with a 3α4β conformation that interacts with the α-ring of the 20S proteasome complex [154,155,156]. The PA28αβ complex binds either to both ends of the 20S proteasome or to only one side of the particle, which binds a 19S regulator on the other side to form the so-called hybrid proteasome (Figure 1B) [147,157,158]. A number of mechanisms have been proposed to explain how PA28αβ increases proteasome activity, including the allosteric activation of the active sites of the proteasome [149] and the stimulation of peptide entry [159] or exit [160,161] from the 20S chamber. Other explanations include the coupling of the proteasome to chaperones that facilitate substrate delivery inside the catalytic chamber or the interaction of PA28-containing proteasomes with ER membranes to facilitate the transfer of the produced peptides into the ER lumen [151,162]. Studying antigen presentation in mice deficient for PA28β, Preckel *et al.* showed that these mice, which lack both PA28α and PA28β, were impaired in their ability to process antigens [163]. In addition, these PA28-deficient cells displayed a dramatic decrease in immunoproteasome content, which, together with the fact that PA28 was found to be associated with immunosubunit-containing hemiproteasomes, led the authors to conclude that PA28αβ was necessary for immunoproteasome assembly [163]. Yet, in subsequent studies using PA28α and PA28β double-knockout mice, Murata *et al.* observed normal immunoproteasome assembly and normal immunity to influenza infection [164]. In this study, however, the authors observed that PA28αβ^−/−^ cells were unable to process a peptide derived from the murine melanoma differentiation antigen, TRP2 (TRP2_181–188_). Overall, the authors concluded that PA28 was not required for proteasome assembly nor overall MHC class I presentation, but was, rather, involved in the processing of a subset of antigenic peptides [164]. Later on, several *in vitro* and *in vivo* studies highlighted a role for PA28αβ in the processing of other MHC class I peptides (Table 2). *In vitro* digestions of precursor peptides by 20S proteasomes in the presence or absence of recombinant PA28αβ showed that PA28αβ increases cleavage efficiency and the production of MHC ligands by the proteasome [165,166]. Association of the proteasome with PA28 also favors double cleavages, which was assumed to originate from the retention by PA28 of the peptide substrate inside the proteasome chamber, thereby increasing the probability for a second cleavage to occur [165]. Transfecting PA28αβ into mouse fibroblasts expressing the murine cytomegalovirus antigen pp89, Groettrup *et al.* observed a marked increase in recognition by the virus-specific cytotoxic T-cells [167]. In this system, presentation of the influenza nucleoprotein was also significantly improved. Later on, the same authors showed that the increase in antigen presentation of the pp89 peptide was independent of the proteasome-subunit composition and therefore, rather, resulted from the general ability of PA28 to activate proteasome complexes without affecting proteasome-subunit composition [168]. Overexpression of PA28αβ also increased the presentation of a number of viral epitopes, while it did not affect the production of others [169,170]. Studying the role of PA28αβ on the processing of the peptide TRP2_360–368_ derived from the human melanoma differentiation antigen, TRP2 [171], Sun *et al.* observed that this peptide was not processed in a melanoma cell line lacking PA28, but this processing was rescued after transient transfection of PA28 cDNAs. Interestingly, the replacement of the flanking sequences of a PA28-independent antigenic peptide by those of the PA28-dependent peptide TRP2_360–368_, revealed that the terminal flanking sequence of the peptide TRP2_360–368_ was sufficient to confer PA28αβ dependency [172]. The origin of this sequence-driven dependency has not yet been clarified. Lastly, an allele-specific involvement of PA28αβ was observed, as it reduced the processing of K^d^ and D^d^ ligands, while favoring the presentation of K^b^ and L^d^ peptides [173]. Overall, PA28αβ appears not to be essential for the MHC class I processing pathway, but rather to be involved in the presentation of a selective set of antigenic peptides.

**Table 2:** The effect on the proteasome regulator PA28αβ on the processing of viral and tumor antigens. LCMV, lymphocytic choriomeningitis virus.

Peptide Source	MHC Restriction	Peptide Sequence	Dependence on PA28αβ	References
**Viral antigens**				
Murine cytomegalovirus pp89_168–176_	H-2L^d^	**YPHFMPTNL**	+	[167,168]
Murine Moloney MuLV Gag-leader_75–83_	H-2D^b^	**CCLCLTVFL**	+	[169]
Murine Moloney MuLV gp75-env_189–196_	H-2K^b^	**SSWDFITV**	−	[169]
LCMV nuceoprotein_118–126_	H-2L^d^	**RPQASGVYM**	−	[174]
JAK1 tyrosine kinase_355–363_	H-2K^d^	**SYFPEITHI**	+	[165]
Influenza A/PR/8 nucleoprotein_146–154_	H-2K^d^	**TYQRTRALV**	+	[167]
**Murine tumor Antigens**				
TRP2_181–188_	H-2K^b^	**VYDFFVWL**	+	[164]
**Human Tumor Antigen**				
TRP2_360–368_	HLA-A2	**TLDSQVMSL**	+	[171,172]
TRP2_288–296_	HLA-A2	**SLDDYNHLV**	−	[172]

#### 4.2.3. The PA28γ

PA28γ was first identified as the *Ki* antigen, target of autoantibodies in the sera of patients with systemic lupus erythematosus [175]. In contrast to PA28αβ, PA28γ is not induced by IFNγ and has a predominant localization in the nucleus [151], where it binds and activates the 20S proteasome as an active homoheptamer [176]. PA28γ-deficient mice display reduced body size and defects in cell proliferation [177,178]. PA28γ-deficient embryonic fibroblasts show defects in mitotic progression and increased apoptosis, suggesting that the regulator is involved in cell cycle progression and apoptosis [177,178]. Accordingly, several binding partners of PA28γ were identified, whose functions are related to apoptosis, such as caspase-7 and MEKK3 [179,180]. Moreover, PA28γ was shown to be involved in the ubiquitin- and ATP-independent degradation of a number of cell-cycle regulatory proteins, such as the cyclin-dependent inhibitor p21, or the steroid receptor coactivator 3 (SRC-3), which is an oncogene often amplified in breast cancer and involved in cell growth [181,182]. Recent studies have also shown that PA28γ is involved in DNA repair and chromosome stability and takes part in the organization of the nuclear speckle and the trafficking of SR proteins (proteins with long serine and arginine repeats, involved in RNA splicing) to the transcription sites [183,184,185]. Interestingly, PA28γ was shown to inhibit the activity of p53, a central transcription factor involved in cell cycle arrest and apoptosis, by inducing p53 nuclear export and promoting its MDM2-dependent ubiquitination and proteasome degradation [186,187]. The role of PA28γ in the stability of p53 was recently shown to originate from the PA28γ-dependent degradation of casein kinase 1 (CK1), which negatively regulates MDM-2 [188]. Additionally, a negative feedback loop between p53 and PA28γ helps with maintaining the balance of p53 and PA28γ in cells [189].

Contrary to PA28αβ, which improves all three catalytic activities of the proteasome; PA28γ was shown to selectively activate the trypsin-like activity without affecting the chymotrypsin-like and the caspase-like activities [190]. However, the role of PA28γ in antigen processing appears relatively insignificant, as PA28γ-deficient mice display normal levels of MHC class I molecules and are not affected in their responses to influenza virus [178]. However, a slower clearance of the intracellular fungal pathogen *Histoplasma capsulatum* was observed, in parallel with a slight decrease in the number of total CD8^+^ T-cells in infected animals [178]. The origin of this defect was not identified.

#### 4.2.4. The PA200 Regulator

PA200 is a 200 kDa protein originally identified in rabbit reticulocyte lysate and bovine testis, where it was bound to 20S proteasomes [148,191]. Similarly to the proteasome regulator, PA28αβ, PA200 stimulates the proteasome hydrolysis of small peptides, but not folded proteins, and it does so in an ATP-independent manner [148]. Contrary to PA28αβ, which activates all three proteasome catalytic activities, or to PA28γ, which activates only the trypsin-like activity, PA200 was shown to increase the hydrolysis of peptides bearing an acidic residue in P1 (caspase-like activity) [148]. PA200 is particularly abundant in testis, where it might play an important role in the maturation of spermatozoa and in the progression of spermatogenesis. Interestingly, after gamma irradiation, cellular levels of PA200/19S hybrid proteasomes are increased [192], and the complex relocalizes to intranuclear foci [148], where PA200 accumulates on chromatin. This leads to an increase in proteasome-related caspase-like activity at the chromatin site [192]. Interestingly, PA200 knockdown was found to impair cell survival after gamma-irradiation [148], a phenomenon that could be counteracted by the addition of exogenous glutamine. This suggested that PA200-deficient cells are unable to cope with the increased glutamine demand that arises following irradiation. The reason for this is not yet clear, but it was suggested that the caspase-like activity of the PA200-19S hybrid proteasome might help with restoring the intracellular levels of glutamine or glutamate necessary for the long-term survival of tumor cells following radiation exposure [192]. So far, no involvement of PA200 in the production of antigenic peptides has been reported.

## 5. Conclusions

The proteasome is a major regulator of protein homeostasis in cells. Through its function in protein degradation, the proteasome enables the cell to adapt to the various assaults and changes occurring in its environment. For each situation, a specific proteasome regulator is involved, granting access to protein substrates into the chamber. Proteasome activity is also regulated by its composition in catalytic subunits, which modulates the cleavage specificities of the particle. As a result of its protease activity, the proteasome releases small peptides that can be loaded onto MHC class I molecules and transported to the cell surface for recognition by the immune system. Proteasome regulation is an important parameter determining the type of peptides presented at the cell surface and thereby affecting the ability of the immune system to respond to the presence of viruses and tumor cells. This occurs at various levels including the establishment of an immunocompetent T-cell repertoire devoid of autoimmune reactivity, the activation of naive T-cells into active effectors cells and the recognition and destruction of tumor or virus-infected cells. Altogether, understanding the mechanisms involved in the production of antigenic peptides by the proteasome is of crucial importance in order to adapt the mode of delivery of immunotherapeutic vaccines and to define which peptides are the most likely to improve the clinical responses to viruses and cancer.
